# Supplementary material for: Mechanism of the small ATP-independent chaperone Spy is substrate specific
Source: Nat Commun. 2021 Feb 8;12:851. doi: 10.1038/s41467-021-21120-8 (PMC7870927; doi:10.1038/s41467-021-21120-8)
Supplement: Supplementary file 1 — Supplementary Information [file 41467_2021_21120_MOESM1_ESM.pdf]

Supplementary Figure 1

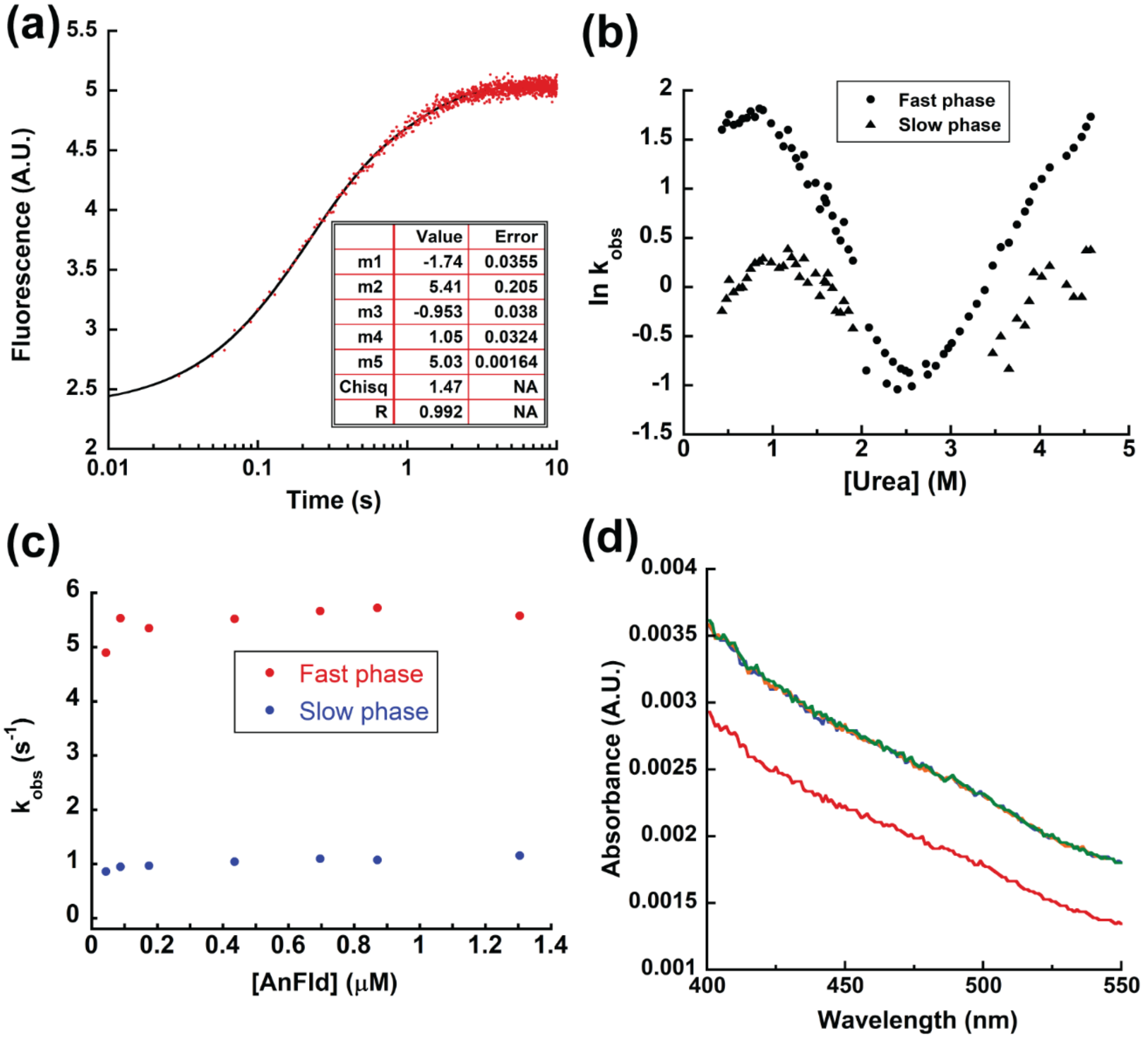

(a) The kinetics of AnFld folding has previously been followed by monitoring its intrinsic tryptophan fluorescence[1]. Fluorescence trace for the kinetic refolding of AnFld monitored by 11.5-fold dilution of 1.04 μM AnFld denatured from HN buffer containing 5 M urea into HN buffer. The average of 10 – 12 traces was fitted to a sum of two exponentials given by the equation $y = m_1e^{-m_2t} + m_3e^{-m_4t} + m_5$ where, y is the fluorescence at time t, m_1 and m_3 are the amplitudes, m_2 and m_4 are the observed rate constants for the major and minor phases respectively, and m_5 is the final fluorescence. A.U., arbitrary units. The black line shows the best fit of the data and the table shown contains the fit parameters. Note the logarithmic scale in the time axis. AnFld's tryptophan fluorescence increased in two kinetic phases: a major (fast) phase with an amplitude of 64.7 % and a minor (slow) phase with an amplitude of 35.3 %. The rate constant for the major phase is 5.4 s^{-1} and that of the minor phase is 1.05 s^{-1}. **(b) - (c)** Before studying the effect that Spy has on AnFld folding, we first wanted to determine if AnFld folds via a similar mechanism in our previously used HN buffer that contains physiological concentrations of NaCl (100 mM), as it does in the 50 mM MOPS buffer used by Fernández-Recio and co-workers that lacks any added salt[1,2]. To get a detailed understanding of AnFld's folding mechanism we followed AnFld folding and unfolding under conditions of varying urea concentration. The

natural logarithms of the folding and unfolding rate constants were plotted as a function of urea concentration to obtain the chevron plot shown in **(b)**. Observation of a logarithmic dependence of both the folding and unfolding rate constants on denaturant concentration would indicate a single transition state barrier with no folding intermediates, i.e. a two-state folding mechanism[3]. Our data, however, reveal a major refolding phase and a slower minor refolding phase; the latter shows a marked curvature (nonlinearity) of the folding limb, meaning that the folding rate at low denaturant concentrations increases with increasing denaturant concentration. The observation of such a "rollover" in the folding arm of the chevron plot is taken as strong evidence for the existence of an off-pathway intermediate[4]. Fernández-Recio and colleagues found that the slow refolding phase does not represent prolyl cis-trans isomerization in the unfolded state and is independent of protein concentration in the range of 1 to 18 μM protein[1]. **(c)** To verify that AnFlavodoxin does not aggregate in our kinetic refolding experiments, we varied the protein concentration (0.5 μM – 15 μM) in the folding experiments. Both the phases were independent of protein concentration. The unfolding kinetics shows two phases, both of which exhibit a linear dependence on urea concentration. While a 3-state linear off-pathway mechanism can adequately explain the two folding phases and the fast unfolding phase, the slow unfolding phase can only be accommodated in a triangular mechanism in the absence of other factors such as aggregation and the purified protein containing a fraction of FMN-bound species (i.e., the holo-protein). **(d)** Absorbance spectra of HN buffer alone (in red) and 100 μM purified AnFld was recorded thrice (in blue, green, and orange). The mean value of absorbance at 450 nm of the protein is 0.0028 A.U., which is similar to the absorbance of buffer alone[5]. The absence of any signal at 450 nm in the absorbance spectrum of the purified protein indicates the absence of holo-protein in the sample. Also, analytical ultracentrifugation confirmed that the purified native protein is monomeric (Figure S3a). Taken together, these observations are indicative of a triangular folding mechanism of AnFld in HN buffer.

Supplementary Figure 2

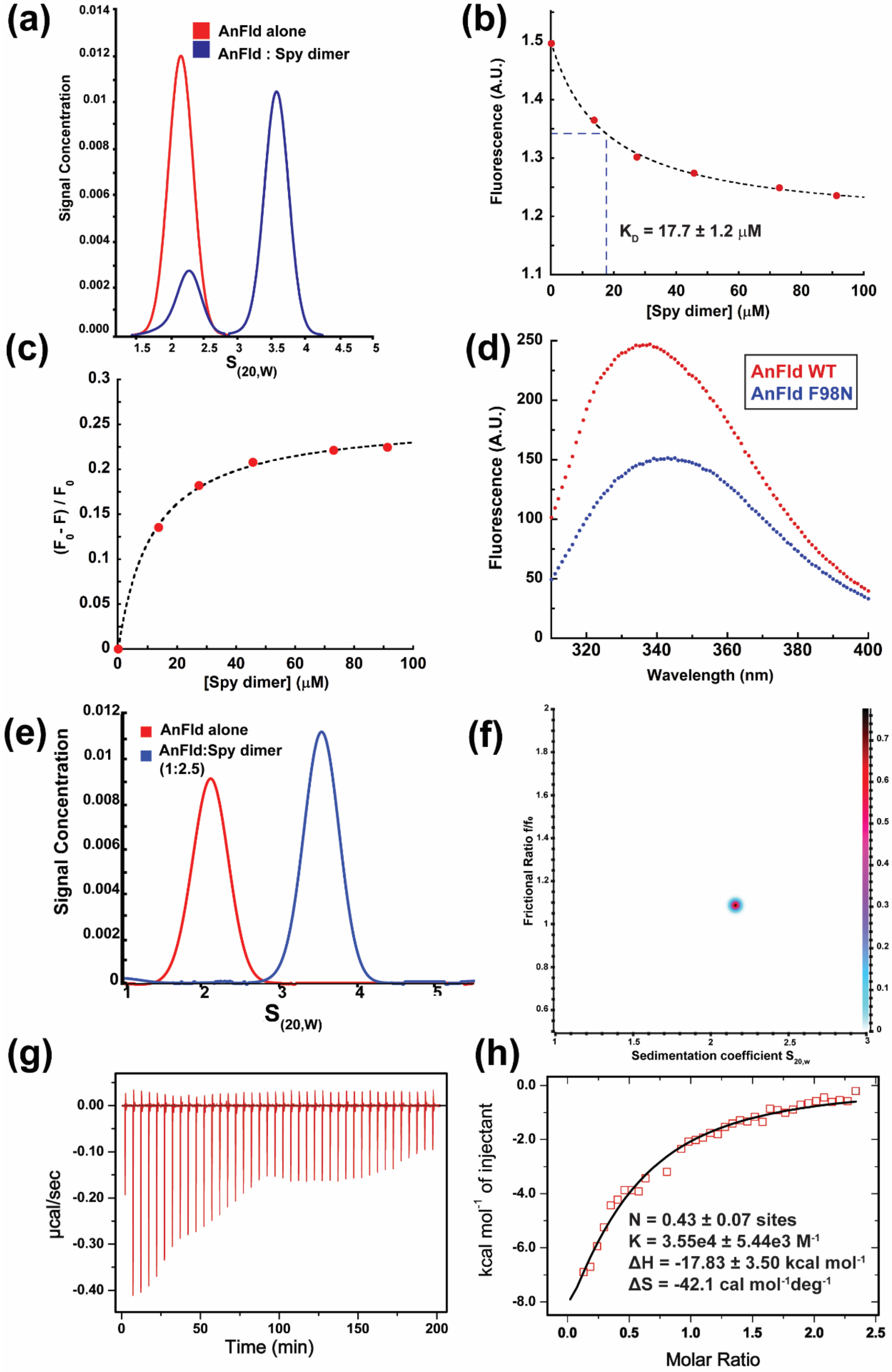

(a) Sedimentation profiles of AnFldWT in the absence (red) or presence of Spy dimer (in 1:1 mole ratio) (blue) from analytical ultracentrifugation experiments performed at 48,000 rpm and 20 °C. The protein samples were

prepared in 10 mM potassium phosphate pH 7.5 buffer, and the concentration of AnFld was 8.8 µM. **(b)** initial and **(c)** final fluorescence was obtained from the fit of the kinetic data sets for the interaction of Spy and 0.09 µM AnFldWT. The plot shows the relative fluorescence signal normalized to fluorescence of AnFldWT alone (F_0) i.e. $\Delta F/F_0$ where $\Delta F = F_0 - F$, as a function of Spy concentration. The black line shows the best fit to a one-site binding model. **(d)** Fluorescence emission spectra of 5 µM each of AnFld WT (red) and F98N (blue) in HN buffer at 25 °C. The excitation wavelength used was 295 nm and emission was monitored from 310 nm to 400 nm. The slit widths were 5 nm each. Note the fluorescence intensity at 340 nm. A.U., arbitrary units. **(e)** Sedimentation of AnFldWT in the absence (red) or presence of Spy dimer (in 1:2.5 mole ratio) (blue) was performed at 48,000 rpm and 20 °C. The protein samples were prepared in 40 mM HEPES-KOH pH 7.5, 25 mM NaCl buffer and the concentration of AnFld was 8.7 µM. **(f)** SV-AUC experiment showing frictional ratios (f/f_0) and sedimentation coefficient for 17.6 µM AnFldWT in 40 mM HEPES-KOH (pH 7.5), 100 mM NaCl at 50,000 rpm and 22 °C. The plot shows the two-dimensional sedimentation analysis (2DSA) followed by analysis with a genetic algorithm, which was further validated by a Monte Carlo analysis. **(g)** – **(h)** ITC data showing the binding of Spy dimer to AnFldWT at 10 °C. The titration syringe was filled with 550 µM Spy dimer and 50 µM AnFldWT was filled in the cell. The thermogram in **(g)** was integrated and fit to a one-site binding model, as shown in **(h)** to obtain thermodynamic binding parameters. N, stoichiometry of binding; K, association constant; ΔH, enthalpy change; ΔS, entropy change. Values reported are the mean ± s.e.m. of the fit.

Supplementary Figure 3

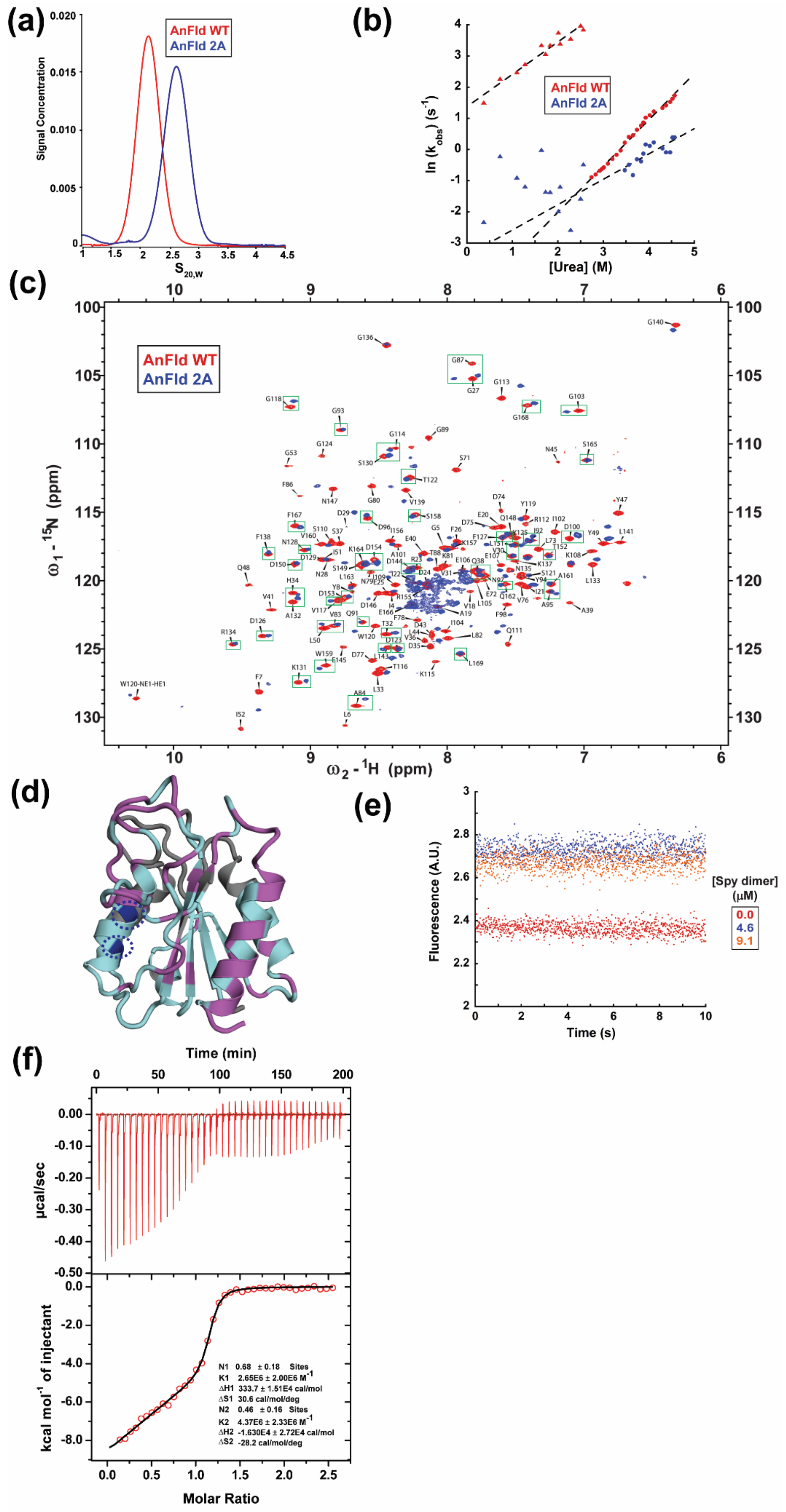

(a) Sedimentation of (17.6 μM each) AnFld WT (red) and 2A mutant (blue) at 50,000 rpm and 22 °C. The protein samples were prepared in HN buffer. **(b)** Major and minor unfolding phases of AnFld WT (red and blue circles respectively) and those of the 2A mutant (red and blue triangles respectively) obtained by fitting the unfolding kinetics in various concentrations of urea to a sum of two exponentials. Black lines show the linear fit of the natural logarithm of k_{obs} for the major unfolding phase as a function of urea concentration according to the equation $ln\ k_{uf} = ln\ k_{uf}^{H_2O} + \frac{m_{uf}}{RT}[Urea]$, where $k_{uf}^{H_2O}$ is the unfolding rate constant in 0 M urea, m_{uf} is the urea-dependence of the unfolding rate constant, R is the gas constant, and T is temperature in Kelvin. The $k_{uf}^{H_2O}$ values for the major unfolding phase of AnFld WT and 2A mutant are 0.007 s^{-1} and 3.96 s^{-1}, respectively. The m_{uf} values for the major unfolding phase of the AnFld WT and 2A mutant are 0.88 kcal mol^{-1} K^{-1} and 0.61 kcal mol^{-1} K^{-1}, respectively. The $k_{uf}^{H_2O}$ and m_{uf} values for the minor phase in AnFld WT unfolding are 0.033 s^{-1} and 0.48 kcal mol^{-1} K^{-1}, respectively. The observation of two unfolding phases for the A2 mutant is consistent with the presence of two populations at equilibrium, in the absence of aggregation. **(c)** 2D [^{1}H-^{15}N] HSQC- TROSY spectra of 0.2 mM AnFld WT (red) and 2A (blue). The cross peaks in the spectra of the 2A mutant that could be assigned unambiguously are shown in green boxes along with the corresponding peak in the WT protein. **(d)** Crystal structure of AnFld WT showing the residues that could be assigned in the 2A mutant (magenta), residues L105 and I109 that are mutated (blue and encircled in blue dotted circles), unassigned residues in the spectra of the mutant (cyan) and residues also unassigned in the WT protein (gray). **(e)** Interaction of AnFld2A mutant and Spy studied in a stopped-flow fluorometer by monitoring tryptophan fluorescence of AnFld following 11.5-fold dilution in HN buffer containing 0 μM (red), 4.6 μM (blue) and 9.1 μM (orange) Spy dimer. **(f)** ITC data showing the binding of Spy dimer to AnFld 2A mutant at 5 °C. The titration syringe was filled with 1.2 mM Spy dimer and 100 μM AnFld2A was filled in the cell. The thermogram was fit to a two-site binding model, which represents two conformations of AnFld 2A in equilibrium that can both bind Spy. The thermodynamic parameters for the two binding species are shown. N, stoichiometry of binding; K, association constant; ΔH, enthalpy change; ΔS, entropy change.

Supplementary Figure 4

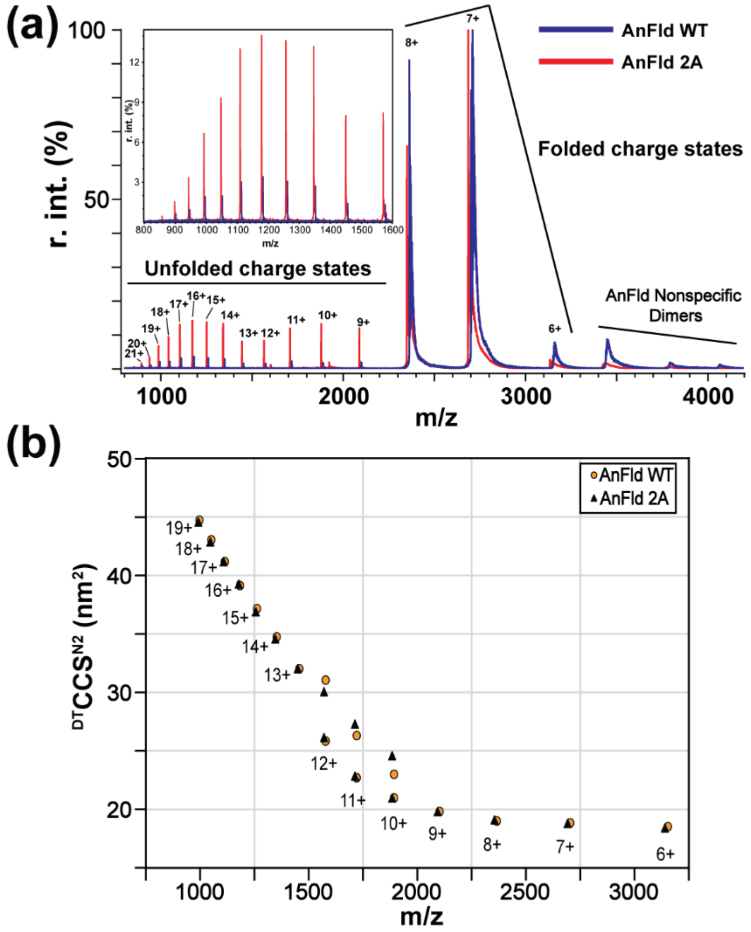

(a) The mass spectra of AnFld WT and AnFld 2A were normalized by intensity and overlaid to compare the charge state distribution, and their relative intensities. The AnFld WT mass spectrum is shown as a black trace, and the AnFld 2A mass spectrum is shown as a red trace. The inset shows the mass range between 800 and 1600 m/z zoomed in. The intensities of these low m/z "unfolded" forms of AnFld 2A are greater than those of AnFld WT indicating that more of AnFld 2A is unfolded in solution. **(b)** The $^{DT}CCS_{N2}$ of AnFld WT and AnFld 2A plotted together to show the similarity in CCS between the two forms of AnFld. The CCSs reported here are available in tabular form in Table S2.

Supplementary Figure 5

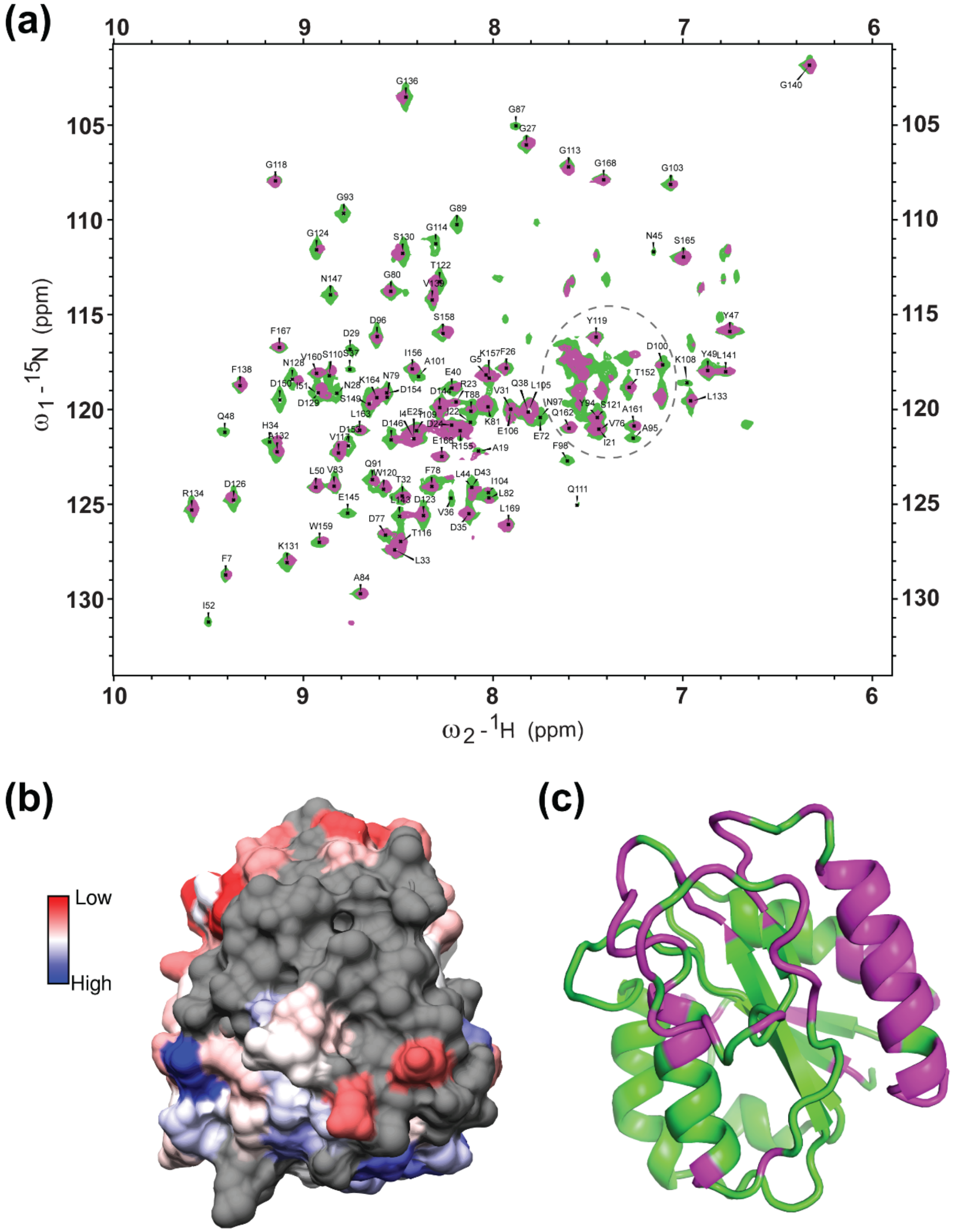

(a) Two-dimensional [^{1}H-^{15}N] HSQC- TROSY NMR spectra of [*U*- ^{2}H, ^{15}N, ^{13}C] -labeled AnFld in the absence (green) and presence of 0.75x Spy (magenta) in 90% (v/v) 50 mM potassium phosphate buffer (pH 7.5) (in $^{1}H_2O$) and 10% (v/v) $^{2}H_2O$. Gray dotted circle shows a region with several overlapping peaks that could not be assigned. 70.5% of the published amide cross peaks of AnFld (BMRB accession number 5011) could be assigned unambiguously in our experiments. **(b)** Structural representation of the backside of the Spy-binding surface in AnFld mapped by NMR peak intensity ratios using a red-to-blue color scale. The unassigned residues are show in gray. Red and blue represent the highest and lowest intensity ratios in the dataset at 0.784 and -0.002 respectively. **(c)** Crystal structure of AnFld showing the residues that interact with Spy in magenta.

Amide cross peaks of these residues have intensity ratios lower than the average value of 0.334. Rest of the protein is colored in green, including residues that are unassigned in our experiments.

Supplementary Figure 6

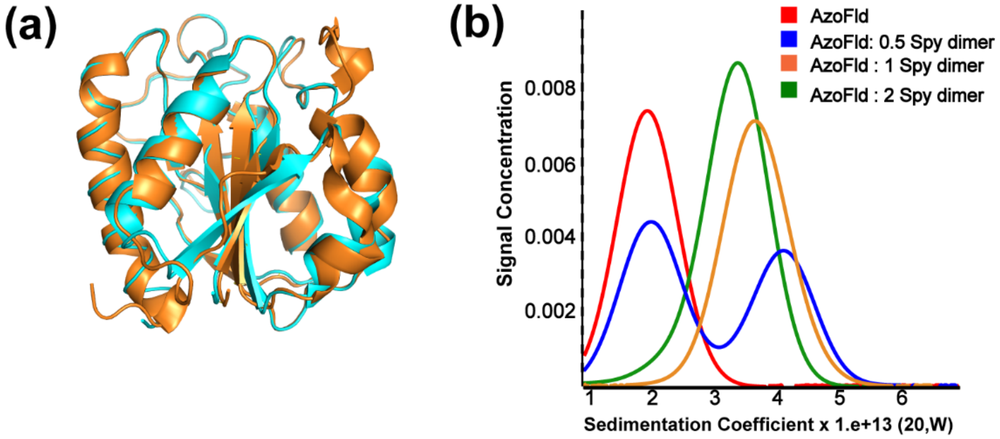

(a) Structural alignment of AnFld (PDB ID 1FTG) (cyan) and AzoFld (PDB ID 1YOB) (orange). **(b)** Samples of AzoFld (13.8 µM) in the absence or presence of 0.5x, 1x and 2x Spy dimer in KP buffer were analyzed by analytical ultracentrifugation at 50,000 rpm and 25°C.

Table S1. Secondary structure determination from buffer-subtracted CD spectra (190-250 nm)

	**Secondary structural element**	**Percentage**
**AnFldWT**	Helix	35.4 %
	Sheet	16.6 %
	Turn	5.3 %
	Others	42.6 %
**AnFld2A**		
	Helix	25.2 %
	Sheet	16.2 %
	Turn	12.3 %
	Others	46.3 %

Table S2

ID	z	**AnFld WT** (18920.4 ± 0.4 Da)			**AnFld 2A** (18,837.0 ± 0.3 Da)		
		**CCS (nm^2)**	**STD**	**R.S.D. (%)**	**CCS (nm^2)**	**STD**	**R.S.D. (%)**
AnFld	19	44.73	0.27	0.60	44.56	0.07	0.16
	18	43.03	0.06	0.15	42.86	0.08	0.19
	17	41.17	0.20	0.49	41.17	0.17	0.42
	16	39.13	0.16	0.41	39.26	0.16	0.40
	15	37.16	0.17	0.47	36.90	0.12	0.32
	14	34.75	0.20	0.58	34.57	0.09	0.25
	13	32.03	0.15	0.48	32.02	0.37	1.15
	12	25.85	0.16	0.63	26.14	0.13	0.50
	12	31.06	0.26	0.83	30.05	0.10	0.33
	11	22.71	0.18	0.78	22.84	0.07	0.30
	11	26.31	0.48	1.81	27.30	0.31	1.13
	10	21.00	0.12	0.58	20.99	0.06	0.29
	10	23.01	0.51	2.20	24.60	0.03	0.14
	9	19.83	0.04	0.19	19.81	0.01	0.06
	8	19.02	0.02	0.12	19.11	0.06	0.30
	7	18.86	0.03	0.14	18.81	0.05	0.27
	6	18.53	0.03	0.14	18.43	0.05	0.27
**ID**	**z**	**Spy-AnFld WT** (50,849 ± 1 Da)			**Spy-AnFld 2A** (50,762 ± 1 Da)		
		**CCS (nm^2)**	**S.D.**	**R.S.D. (%)**	**CCS (nm^2)**	**S.D.**	**R.S.D. (%)**
Spy-AnFld	13	36.79	0.02	0.05	36.51	0.06	0.16
	12	36.39	0.07	0.19	35.91	0.06	0.16
	11	35.94	0.06	0.16	35.50	0.08	0.22
	10	35.79	0.19	0.54	35.26	0.05	0.14
**ID**	**z**	**Spy dimer** (31,931 ± 2 Da)					
		**CCS (nm^2)**	**S.D.**	**R.S.D. (%)**			
Spy dimer	11	26.29	0.08	0.30			
	10	25.65	0.15	0.59			
	9	24.87	0.21	0.83			
	8	23.81	0.21	0.89			

Table S3

Primer	Mutation	Sequence (5′ to 3′)
RM01	L105A and I109A	CACGCTGGCTAGCCTTTTCCTCCGCAATGCCGATCGCGTCTTGGA
RM02	L105A and I109A	TCCAAGACGCGATCGGCATTGCGGAGGAAAAGGCTAGCCAGCGTG
RM03	F98N	CGGTTATGCGGATAACAACCAAGACGCGATCGGC
RM04	F98N	GCCGATCGCGTCTTGGTTGTTATCCGCATAACCG

References

1. Fernández-Recio, J., Genzor, C. G. & Sancho, J. Apoflavodoxin folding mechanism: An α/β protein with an essentially off-pathway intermediate. *Biochemistry* **40**, 15234–15245 (2001).
2. Stull, F., Koldewey, P., Humes, J. R., Radford, S. E. & Bardwell, J. C. A. Substrate protein folds while it is bound to the ATP-independent chaperone Spy. *Nat. Struct. Mol. Biol.* **23**, 53–58 (2016).
3. Brockwell, D. J. & Radford, S. E. Intermediates: ubiquitous species on folding energy landscapes? *Curr. Opin. Struct. Biol.* **17**, 30–37 (2007).
4. Baldwin, R. L. On-pathway versus off-pathway folding intermediates. *Fold. Des.* **1**, (1996).
5. Klugkist, J., Voorberg, J., Haaker, H. & Veeger, C. Characterization of three different flavodoxins from Azotobacter vinelandii. *Eur. J. Biochem.* **155**, 33–40 (1986).
