# Supplementary figures and images for: Mechanism of the small ATP-independent chaperone Spy is substrate specific
Source: Nat Commun. 2021 Feb 8;12:851. doi: 10.1038/s41467-021-21120-8 (PMC7870927; doi:10.1038/s41467-021-21120-8)

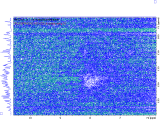

Supplement: Supplementary file 5 — Supplementary Data 3 [file 41467_2021_21120_MOESM5_ESM.zip › AnFld WT and 2A spectra/ANFLD-A2/11/pdata/1/thumb.png]

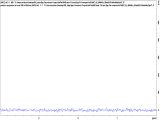

Supplement: Supplementary file 5 — Supplementary Data 3 [file 41467_2021_21120_MOESM5_ESM.zip › AnFld WT and 2A spectra/ANFLD-A2/11/pdata/999/thumb.png]

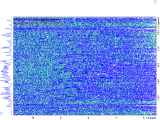

Supplement: Supplementary file 5 — Supplementary Data 3 [file 41467_2021_21120_MOESM5_ESM.zip › AnFld WT and 2A spectra/ANFLD-WT/21/pdata/1/thumb.png]

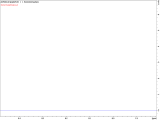

Supplement: Supplementary file 5 — Supplementary Data 3 [file 41467_2021_21120_MOESM5_ESM.zip › AnFldWT-Spy interaction/AnFld/pdata/1/thumb.png]

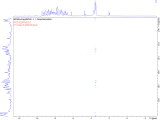

Supplement: Supplementary file 5 — Supplementary Data 3 [file 41467_2021_21120_MOESM5_ESM.zip › AnFldWT-Spy interaction/AnFld_Spy/pdata/1/thumb.png]
