# Supplementary material for: Mechanism of the small ATP-independent chaperone Spy is substrate specific
Source: Nat Commun. 2021 Feb 8;12:851. doi: 10.1038/s41467-021-21120-8 (PMC7870927; doi:10.1038/s41467-021-21120-8)
Supplement: Supplementary file 6 — Description of additional supplementary files [file 41467_2021_21120_MOESM6_ESM.docx]

Supplementary Dataset 1. AUC data

Supplementary Dataset 2. ITC data

Supplementary Dataset 3. NMR data
